# Pet Animals Were Infected with SARS-CoV-2 from Their Owners Who Developed COVID-19: Case Series Study

**DOI:** 10.3390/v15102028

**Published:** 2023-09-29

**Authors:** Yudai Kuroda, Kei Watanabe, Tsukasa Yamamoto, Hiroki Suzuki, Eun-sil Park, Keita Ishijima, Kango Tatemoto, Milagros Virhuez-Mendoza, Yusuke Inoue, Michiko Harada, Ayano Nishino, Tsuyoshi Sekizuka, Makoto Kuroda, Tsuguto Fujimoto, Genki Ishihara, Ryo Horie, Kosuke Kawamoto, Ken Maeda

**Affiliations:** 1 Department of Veterinary Science, National Institute of Infectious Diseases (NIID), Toyama, Shinjuku-ku, Tokyo 162-8640, Japan; 2 Anicom Specialty Medical Institute Inc., Nishi-shinjuku, Shinjuku-ku, Tokyo 160-0023, Japan; 3 Joint Graduate School of Veterinary Medicine, Yamaguchi University, Yoshida, Yamaguchi 753-8515, Japan; 4 Pathogen Genomics Center, NIID, Toyama, Shinjuku-ku, Tokyo 162-8640, Japan; 5 Department of Fungal Infection, NIID, Toyama, Shinjuku-ku, Tokyo 162-8640, Japan

**Keywords:** SARS-CoV-2, cats, dogs

## Abstract

Severe acute respiratory syndrome coronavirus 2 (SARS-CoV-2) infection among pets owned by coronavirus disease 2019 (COVID-19) patients has been reported around the world. However, how often the animals are exposed to SARS-CoV-2 by their owners is still unclear. We have collected swab samples from COVID-19 patients’ pets and performed real-time RT-PCR to detect the viral genome. In total, 8 of 53 dogs (15.1%) and 5 of 34 cats (14.7%) tested positive for the SARS-CoV-2 N gene. The result of a virus neutralization (VN) test also showed VN antibodies in four cats and six dogs. Our results indicate that the virus often passed from infected owners to their pets, which then excreted the virus despite having no or mild clinical signs.

## 1. Introduction

Severe acute respiratory syndrome coronavirus 2 (SARS-CoV-2) is an emerging pathogen of coronavirus disease 2019 (COVID-19) with a broad host range [1]. Under laboratory conditions, hamsters, cats and ferrets are likely to be more susceptible to SARS-CoV-2 than dogs, and pigs and chickens are not susceptible [2]. In addition, SARS-CoV-2 natural infections in companion animals, such as dogs and cats, have been reported worldwide, and the main transmission route is thought to be through the owners [3,4,5]. Recently, SARS-CoV-2 was transmitted from an infected cat to a veterinarian in Thailand [6]. To prevent SARS-CoV-2 transmission from infected pets to humans, it is necessary to clarify the SARS-CoV-2 infection rate among pets owned by COVID-19 patients. In this study, we examined the natural transmission frequency of SARS-CoV-2 from patients to their pets.

## 2. Materials and Methods

### 2.1. Cells and Viruses

VeroE6/TMPRSS2 cells [7] (JCRB Cell Bank no. JCRB1819) were maintained in Dulbecco’s modified Eagle’s medium (DMEM; SIGMA-ALDRICH, St. Louis, MO, USA) supplemented with 10% fetal bovine serum (FBS; SIGMA-ALDRICH), 1% penicillin-streptomycin (FUJIFILM Wako Pure Chemical Corporation, Osaka, Japan) and 1 mg/mL G-418 (Roche Diagnostics, Basel, Switzerland) at 37 °C with 5% CO_2_. SARS-CoV-2 WK-521 (Wuhan), TY26-439 (Delta variant) and TY38-873 (Omicron BA.1 variant) were propagated in VeroE6/TMPRSS2 and stored at −80 °C until use for virus neutralization (VN) tests. Nucleotide sequences data of three strains are available from GISAID [8] (WK-521: EPI_ISL_408667; TY26-439: EPI_ISL_3374455 and TY38-873: EPI_ISL_7418017).

### 2.2. Sample Collection

In Japan, many COVID-19 patients were hospitalized [9], and therefore could not care for their pets during the hospitalization period. Anicom Advanced Medical Research Institute Co., Ltd. organized a volunteer program to take care of pets, regardless of their clinical signs. Just before their owners were hospitalized after being diagnosed with COVID-19 by their doctor, the animals were taken to a shelter, and at least oral swabs were collected using cotton swabs. Swab samples were collected from 53 dogs, 34 cats, 2 rabbits, and 1 hedgehog from 10 April 2020, to 23 April 2022, before the animals were placed in their care, and the samples were mixed with PBS or virus preservation solution (Sugiyama-Gen. Co., Ltd., Tokyo, Japan). Many dogs and cats were kept alone in households, and some of them were kept in the same house. Subsequently, the collected samples were tested for SARS-CoV-2 at the institute via real-time RT-PCR. Positive samples were confirmed and further analyzed at the Department of Veterinary Science, National Institute of Infectious Diseases in Japan. All SARS-CoV-2-positive animals were isolated from the other animals, and then samples were collected serially. When the test was negative on two consecutive days, the animals were moved to a regular area. In addition to swab samples, blood samples were also collected from positive animals and centrifuged at 800× *g* for 5 min. The serum samples were stored at 4 °C until use for the VN test.

### 2.3. Virus Isolation

Swab samples were mixed with PBS or virus preservation solution (Sugiyama-Gen. Co., Ltd., Tokyo, Japan) and centrifuged at 1110× *g* for 5 min at 4 °C. The supernatants were filtered through 0.45 µm filters (Corning, Corning, NY, USA). VeroE6/TMPRSS2 cells were inoculated with the filtrates and incubated at 37 °C overnight. After the incubation, the culture medium was replaced with fresh DMEM containing 2% FBS and antibiotics. The cells were incubated until cytopathic effect (CPE) was observed. All procedures were performed at the biosafety level 3 facilities at NIID.

### 2.4. Real-Time Reverse Transcription-Polymerase Chain Reaction (RT-PCR)

First, RNA detection was carried out at Anicom Advanced Medical Research Institute Co., Ltd. with real-time RT-PCR using the SARS-CoV-2 Detection Kit-N2 set-(TOYOBO, Osaka, Japan) and QuantStudio 7 Flex (Thermo Fisher, Waltham, MA, USA).

The quantification of viral RNA in swab samples with real-time RT-PCR assays was performed at NIID in Japan, according to our previous report [10]. Briefly, swab samples in PBS were centrifuged at 1110× *g* for 5 min at 4 °C, and RNA was extracted from the supernatants using the QIAamp Viral RNA Mini Kit (QIAGEN, Hilden, Germany). A real-time RT-PCR assay was conducted using LightCycler 480 (Roche Diagnostics). Primer set N2 was used in this assay. The RT-PCR cycling protocol was as follows: 50 C for 30 min; 95 °C for 15 min; and 45 cycles of 95 °C for 15 s and 60 °C for 60 s. To determine the number of RNA copies, a positive control was diluted 10-fold, and the final concentration was from 50,000 copies/well to 50 copies/well. The nucleotide sequence of the positive control is shown in a previous report [10]. Samples were judged as positive for SARS-CoV-2 RNA if the Ct values were less than 40.

### 2.5. VN Test

A monolayer of VeroE6/TMPRSS2 cells was prepared on a 96-well microplate (IWAKI, Tokyo, Japan). Complement in serum samples was inactivated at 56 °C for 30 min, and then sera were diluted with DMEM containing 10% FBS via 2-fold serial dilution. The diluted serum was mixed with the same volume of 100 TCID_50_/50 µL of SARS-CoV-2. After a neutralization reaction at 37 °C for 1 h, 100 µL of the mixture was transferred to each well in the 96-well microplate. The cells were incubated at 37 °C for 5 days, fixed with 10% buffered formalin (FUJIFILM Wako Pure Chemical Corporation) and stained with methylene blue to observe CPE. The neutralization titer was defined as the highest dilution at which 50% CPE inhibition was observed.

### 2.6. Sequence Analysis

Complete genome sequencing was performed through a combination of multiplex PCR and next-generation sequencing (NGS), referring to ARTIC Network’s modified protocol [11]. Reverse transcription was carried out using a LunaScript RT SuperMix Kit (New England Biolabs, Ipswich, MA, USA). The temperature profile was 2 min at 25 °C, 20 min at 55 °C and 1 min at 95 °C. Then, a multiplex PCR reaction was performed using a Q5 High-Fidelity DNA Polymerase (NEW ENGLAND Biolabs) with the following cycling protocol: 1 cycle at 98 °C for 30 s, and 30 cycles at 98 °C 15 s and 65 °C for 5 min. The PCR products were purified using an Agencourt AMPure XP (Beckman Coulter, Inc., Brea, CA, USA). NGS libraries were constructed using a QIAseq FX DNA Library Kit (QIAGEN). After the libraries were pooled and purified, they were sequenced using a MiSeq (Illumina, Inc., San Diego, CA, USA) or MiSeq v2 Reagent Kit (Illumina, Inc.). All reads were mapped to the reference sequence of the SARS-CoV-2 Wuhan-Hu-1 strain (GenBank: MN908947.3) by *bwa mem* [12]. The consensus sequence was extracted using the CLC Genomics Workbench (QIAGEN). Sanger sequencing was carried out by Eurofins Genomics, Japan, to validate the region where the coverage was insufficient. All of the whole-genome sequences determined in this research were deposited in GISAID.

## 3. Results

### 3.1. Viral RNA Detection

In total, five cats (14.7% [95% CI 6.8–27.6%]) and eight dogs (15.1% [95% CI 5.0–31.1%]) were confirmed to be positive for SARS-CoV-2 via real-time RT-PCR; the characteristics of these animals are shown in Table 1. Four cats were of mixed breeds, and one cat was a Ragdoll, while three dogs were Toy Poodles, two dogs were Shiba and the remaining three dogs were a Chihuahua, a Cairn Terrier and a dog of mixed breed. VetC1 and VetC2, VetC6 and VetC7, and VetD3 and VetD4 came from the same households. Quantification of viral RNA showed that two cats, VetC1 and VetC7, and two dogs, VetD5 and VetD12, had high copy numbers reaching over 10,000 copies per reaction, and viral RNA shedding continued up to sampling day 15 (Day 15) in the cat VetC7 and up to Day 11 in the dog VetD11 (Table 2). Virus isolation was successful using an oral swab for VetC1. One cat (VetC8) exhibited nasal discharge, and one dog (VetD12) exhibited loose stool. The other dogs and cats did not show any apparent clinical signs (Table 1).

### 3.2. Detection of VN Antibodies against Three Diffenrent Variants of SARS-CoV-2

Serum samples were collected from the SARS-CoV-2-positive animals, except for VetD5, for the virus neutralization (VN) test for SARS-CoV-2 [13,14]. The VN test is the gold standard for detecting antibodies, and it has been reported to be a more specific method than enzyme-linked immunosorbent assays for detecting N in cats [15]. WK-521, TY26-439 and TY38-873 were examined as representative viruses of the Wuhan, Delta and Omicron BA.1.18 variants, respectively. The seropositive cats (four of the five SARS-CoV-2-positive cats) possessed VN antibodies against all three variants. Among the seropositive dogs (six of the seven SARS-CoV-2-positive dogs included in this analysis), five dogs (83.3%) possessed VN antibodies against the Wuhan variant, six dogs (100%) possessed VN antibodies against the Delta variant and only one dog (16.7%) possessed VN antibodies against the Omicron variant (Table 3).

### 3.3. Complete Genome Sequencing

We determined the nearly complete nucleotide sequences of SARS-CoV-2 in five dogs and two cats with next-generation sequencing and Sanger sequencing (Table 4). These viruses from VetC1 and VetC7 were classified into B.1.1.214, those from VetD2, VetD3 and VetD5 were classified into B.1.1.284, and those from VetD12 and VetD15 were into AY.29 (Delta) with a Pangolin tool.

## 4. Discussion

In this study, SARS-CoV-2 RNA was detected in 5 of 34 cats and 8 of 53 dogs owned by patients with COVID-19. Of these SARS-CoV-2-infected animals, only one cat and one dog showed clinical signs, i.e., mild signs of nasal discharge and loose stool, respectively. We could not confirm whether these clinical signs were caused by SARS-CoV-2 infection, but based on our findings, we suspect that many dogs and cats are infected with SARS-CoV-2 without obvious signs. Although the clinical signs seen in this study were mild, in August 2021, we found a cat with severe respiratory signs caused by the SARS-CoV-2 Delta variant in Japan [16].

The detection rate of SARS-CoV-2 RNA was 15.1% in dogs and 14.7% in cats, indicating that domestic dogs and cats in COVID-19-positive households are exposed to SARS-CoV-2 from their owners at a similar frequency. Previous reports have shown that the detection rate of SARS-CoV-2 RNA in the pets of patients with COVID-19 ranged from 1.7% to 26.2% in dogs in Ecuador and the United States, and 12.0% to 17.6% in cats in Ecuador, the United States and China [17,18,19]. In the present study, the detection rate in cats was similar to the previously reported rates, but that in dogs differed. Under experimental conditions, the period of viral RNA shedding was reported to be shorter in dogs than in cats [2]. Our SARS-CoV-2 RNA detection rates in dogs were higher than those in the previous report, likely because sampling was performed just before the hospitalization of the pets’ owners. From our results, it appears that SARS-CoV-2 is transmitted from patients to approximately 15% of the companion animals in COVID-19-positive households. To prevent their pets from SARS-CoV-2 infection, patients should not have close contact with the animals, such as kissing or sharing food [20]. Many dogs and cats may have been infected with SARS-CoV-2 in Japan up to now, and some of them may have shown clinical signs and were brought to a veterinary hospital. SARS-CoV-2 transmission from patients to minks and from infected minks to humans have been reported [20]. Veterinarians and their associates should thus be careful regarding the possibility of reverse infection with SARS-CoV-2 from diseased animals to humans.

The serum of all seropositive cats could neutralize the Wuhan, Delta and Omicron variants of SARS-CoV-2, but only one of the six seropositive dogs had antibodies against all of these variants (Table 3). The VN antibodies induced in cats might be broader than those in dogs. Therefore, for serological diagnosis and surveillance, suitable SARS-CoV-2 variants must be selected for VN testing, especially in dogs.

Several mutations in the viral proteins of SARS-CoV-2 have been reported in animal infections. For example, amino acid substitutions F486L and N501T in the spike protein were commonly observed in mink-associated SARS-CoV-2, and the virus showed increased binding affinity for mink angiotensin-converting enzyme 2 [21]. In addition, N501T led to a higher binding affinity for human angiotensin-converting enzyme 2 [21]. Recently, the same mutations were identified in the viral sequences detected in white-tailed deer in Ontario, Canada [22]. Therefore, the variants that emerge in animals may possess higher infectivity in animals. Further investigations in animals are required to understand the mutations that emerge during infections among animals.

## 5. Conclusions

Our results indicated that SARS-CoV-2 transmission from humans to cats and dogs occurs at a high frequency. Although many SARS-CoV-2-infected cats and dogs did not show clinical signs, some of them did. Since virus transmission to different animals might induce mutations for adaptation to the animals, the transmission of SARS-CoV-2 from owners to pets must be prevented.

## Figures and Tables

**Table 1:** Characteristics of the SARS-CoV-2-positive cats and dogs in this study.

Species	Animal ID	Date of Visit (y/m/d)	Age	Sex	Breed	Clinical Sign
Cat	VetC1	2020/9/12	1 year	Male	Mixed	No
	VetC2	2020/9/12	1 year	Female	Mixed	No
	VetC6	2021/1/13	9 years	Female	Ragdoll	No
	VetC7	2021/1/13	9 years	Female	Mixed	No
	VetC8	2021/1/30	11 years	Female	Mixed	Nasal discharge
Dog	VetD2	2020/7/26	11 months	Male	Chihuahua	No
	VetD3	2020/7/31	1 year	Male	Shiba	No
	VetD4	2020/7/31	2–3 years	Female	Toy Poodle	No
	VetD5	2020/8/7	17 years	Female	Cairn Terrier	No
	VetD10	2021/2/11	1 year	Female	Shiba	No
	VetD11	2021/7/9	8 years	Female	Toy Poodle	No
	VetD12	2021/8/1	1 year	Male	Mixed	Loose stool
	VetD15	2021/9/1	5 years	Male	Toy Poodle	No

**Table 2:** Detection of SARS-CoV-2 genes in the cat and dog samples via real-time RT-PCR.

Animal ID	Date on Day 1 (y/m/d)	Swab Sample	Day																
			1	2	3	4	5	6	7	8	9	10	11	12	13	14	15	16	17
VetC1	2020/9/12	O	2635 ^a^	6410	29,550	2205	185	10,200	3525	-	-	-	+	-	-				
VetC2	2020/9/12	O	+ ^b^	+	+	- ^c^	-	+	+	-	-	-	-	-	-	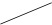	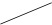	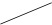	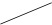
VetC6	2021/1/13	P	+	+	-	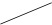	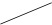	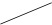	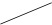	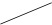	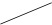	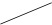	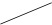	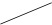	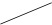	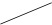	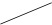	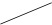	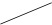
		O	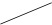	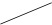	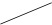	-	-	-	-	-	-	-	-	-	-	-	-	-	-
VetC7	2021/1/13	P	50,000<	609	212	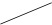	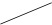	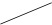	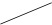	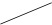	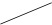	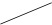	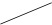	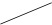	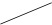	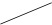	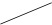	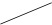	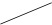
		O	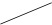	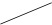	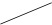	+	-	-	+	-	+	+	-	+	1063	+	+	-	-
VetC8	2021/1/30	O	64	295	+	-	+	-	+	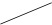	-	190	-	-	-	-	-	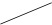	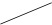
VetD2	2020/7/26	P	+	1855	-	-	-	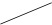	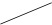	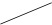	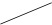	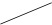	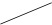	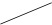	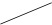	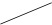	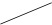	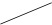	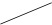
		F	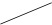	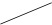	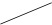	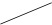	-	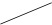	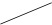	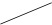	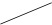	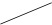	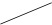	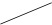	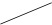	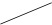	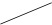	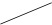	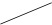
		U	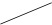	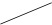	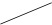	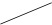	-	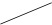	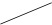	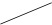	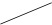	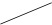	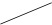	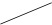	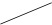	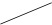	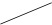	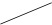	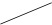
VetD3	2020/7/31	P	195	3325	733	-	-	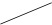	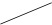	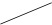	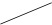	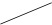	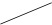	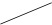	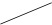	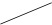	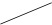	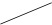	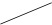
VetD4	2020/7/31	P	+	-	-	-	-	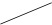	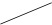	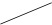	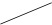	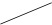	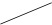	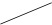	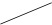	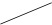	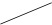	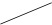	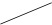
VetD5	2020/8/7	P	8545	22,050	47,400	12,950	14,450	1175	+	+	+	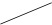	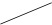	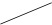	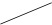	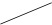	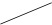	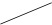	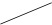
		O	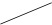	5770	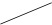	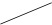	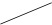	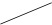	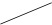	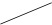	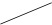	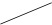	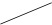	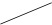	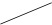	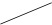	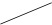	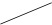	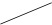
		F	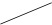	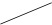	-	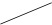	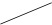	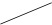	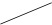	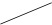	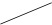	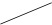	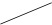	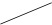	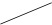	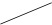	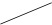	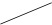	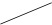
VetD10	2021/2/11	O	-	-	-	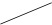	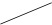	530	+	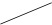	-	-	-	-	-	-	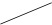	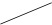	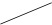
VetD11	2021/7/9	O	1430	1270	7270	2450	1290	328	635	+	-	+	72	-	-	-	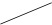	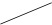	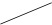
VetD12	2021/8/1	O	+	-	+	11,500	2440	+	-	+	-	-	-	-	-	-	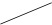	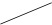	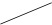
VetD15	2021/9/1	O	+	2200	-	-	-	-	-	-	-	-	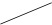	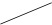	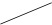	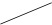	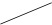	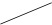	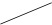

^a^: Numbers indicate the copy number of SARS-CoV-2 RNA per reaction; ^b^: “+” indicates that the sample was positive according to real-time RT-PCR, but the copy number was less than 50 copies per reaction; ^c^: “-” indicates a value under the detection limit; O: oral swab; N: nasal swab; P: pharyngeal swab; R: rectal swab; F: fecal swab; U: urine.

**Table 3:** Virus neutralization titer of 5 cats and 7 dogs against the SARS-CoV-2 WK-521, TY26-439 and TY38-873 variants.

Species	Animal ID	Day	VN Titer		
			WK-521 (Wuhan)	TY26-439 (Delta)	TY38-873 (Omicron)
Cat	VetC1	16	450	N.D.	N.D.
		32	127	80	40
	VetC2	16	110	N.D.	N.D.
		32	**64**	**40**	10
	VetC6	21	<5	<5	<5
	VetC7	21	**80**	**40**	10
	VetC8	7	**160**	**80**	5
		14	**80**	**80**	10
		21	80	80	40
Dog	VetD2	4	<5	N.D.	N.D.
		14	<5	N.D.	N.D.
		28	5	5	<5
	VetD3	14	<5	**20**	<5
		31	**40**	**20**	5
	VetD4	14	<5	<5	<5
		31	<5	<5	<5
	VetD10	23	10	10	<5
	VetD11	7	<5	<5	<5
		14	<5	**80**	<5
		22	<5	**80**	<5
		30	<5	**80**	<5
	VetD12	7	<5	**20**	<5
		14	10	**80**	<5
		21	10	**80**	<5
		28	10	**40**	<5
	VetD15	14	5	**20**	<5

Bold letters indicate 4-fold higher VN titers than those against Omicron variant. N.D.: not determined.

**Table 4:** Sequence data of SARS-CoV-2 in dogs and cats.

Species	Animal ID	Sample	Date of Sample Collection	GISAID ID	Pango Lineage
Dog	VetD2	Pharyngeal swab	27 July 2020	EPI_ISL_14436255	B.1.1.284
	VetD3	Pharyngeal swab	1 August 2020	EPI_ISL_14436256	B.1.1.284
	VetD5	Pharyngeal swab	9 August 2020	EPI_ISL_14436257	B.1.1.284
	VetD12	Oral swab	4 August 2021	EPI_ISL_14436253	AY.29
	VetD15	Oral swab	4 September 2021	EPI_ISL_14436254	AY.29
Cat	VetC1	Isolated virus (Oral swab)	14 September 2020	EPI_ISL_1358217	B.1.1.214
	VetC7	Pharyngeal swab	13 January 2021	EPI_ISL_1358218	B.1.1.214

## Data Availability

The data shown in this research are available in the article.
